# Testing the Use of Data Drawn from the Electronic Health Record to Compare Quality

**DOI:** 10.1097/pq9.0000000000000432

**Published:** 2021-07-28

**Authors:** Kathleen E. Walsh, Hanieh Razzaghi, David M. Hartley, Levon Utidjian, Shannon Alford, Rahul A. Darwar, Elizabeth Shenkman, Susannah Jonas, Mary Goodick, Jonathan Finkelstein, Al Ozonoff, L. Vandy Black, Michael Shapiro, Kathryn Shaw, Jennifer McCafferty-Fernandez, Keith Marsolo, Amy Kelly, Lloyd N. Werk, Jordan Smallwood, Charles Bailey

**Affiliations:** From the * Boston Children’s Hospital, Boston, Mass.; † Department of Pediatrics, Harvard Medical School, Boston, Mass.; ‡ Department of Pediatrics, Children’s Hospital of Philadelphia, Philadelphia, Pa.; § Division of Allergy-Immunology, Nemours Children’s Hospital, Orlando, Fla.; ¶ James M. Anderson Center for Health Systems Excellence, Cincinnati Children’s Hospital, Cincinnati, Ohio; ∥ Department of Pediatrics, University of Cincinnati, College of Medicine, Cincinnati, Ohio; ** Department of Biomedical and Health Informatics, Children’s Hospital of Philadelphia, Philadelphia, Pa.; †† Department of Health Outcomes and Biomedical Informatics, University of Florida, College of Medicine, Gainesville, Fla.; ‡‡ Division of Pediatric Hematology and Oncology, University of Florida College of Medicine, Gainesville, Nicklaus Children’s Hospital, Miami, Fla.; §§ Department of Psychiatry, University of Florida College of Medicine, Gainesville, Fla.; ¶¶ Nicklaus Children’s Health System, Miami, Fla.; ∥∥ Department of Population Health Sciences, Duke University School of Medicine, Durham, N.C.; *** Devereux Advanced Behavioral Health, Berwyn, Pa.; ††† Division of General Academic Pediatrics, Nemours Children’s Hospital, Orlando, Fla.; ‡‡‡ Department of Pediatrics, Perelman School of Medicine, University of Pennsylvania, Philadelphia, Pa.

## Abstract

**Introduction:**

Health systems spend $1.5 billion annually reporting data on quality, but efficacy and utility for benchmarking are limited due, in part, to limitations of data sources. Our objective was to implement and evaluate measures of pediatric quality for three conditions using electronic health record (EHR)-derived data.

**Methods:**

PCORnet networks standardized EHR-derived data to a common data model. In 13 health systems from 2 networks for 2015, we implemented the National Quality Forum measures: % children with sickle cell anemia who received a transcranial Doppler; % children on antipsychotics who had metabolic screening; and % pediatric acute otitis media with amoxicillin prescribed. Manual chart review assessed measure accuracy.

**Results:**

Only 39% (N = 2,923) of 7,278 children on antipsychotics received metabolic screening (range: 20%–54%). If the measure indicated screening was performed, the chart agreed 88% of the time [95% confidence interval (CI): 81%–94%]; if it indicated screening was not done, the chart agreed 86% (95% CI: 78%–93%). Only 69% (N = 793) of 1,144 children received transcranial Doppler screening (range across sites: 49%–88%). If the measure indicated screening was performed, the chart agreed 98% of the time (95% CI: 94%–100%); if it indicated screening was not performed, the chart agreed 89% (95% CI: 82%–95%). For acute otitis media, chart review identified many qualifying cases missed by the National Quality Forum measure, which excluded a common diagnostic code.

**Conclusions:**

Measures of healthcare quality developed using EHR-derived data were valid and identified wide variation among network sites. This data can facilitate the identification and spread of best practices.

## INTRODUCTION

Physician practices in the United States spend approximately $1.5 billion annually reporting healthcare quality measures for benchmarking; data entry accounts for most of this cost.^1^ Unfortunately, 70% do not use data reported to inform quality improvement activity.^1^ Benchmarks are standardized measures used to compare the quality of healthcare received from different clinics or facilities.^2^ Benchmarking is an essential and integral component of continuous quality improvement in healthcare.^3^ The concern that reporting quality of care data to payers, regulatory agencies, consumer reporting agencies, and others is costly, and does not contribute to healthcare quality improvement which is widely recognized in the literature.^4–6^ Nonetheless, health systems, clinicians, and consumers need valid, actionable, efficient benchmarking to improve healthcare quality and safety.^7^

For transformational rather than incremental improvement in healthcare quality, several changes, anchored to the National Quality Forum (NQF) management of a set of measures, are required, including reduced reliance on claims-based measures through the transition to measures using electronic health record (EHR) data, generated through care delivery.^7^ The NQF is a public–private partnership that helps determine the best measures for evaluating and rewarding safe, high-quality healthcare.^8^ Benchmarking, based on data drawn directly from the EHR, offers advantages relative to other sources due to their timeliness, accuracy, and digital (ie, computable) form.^9^ The addition of electronic clinical quality measure specifications to the NQF-endorsed measures is intended to address problems of scalability and automation seen with measures that rely on manual chart review and abstraction. However, hospitals need to map the electronic specifications to the data structures commonly found in EHRs.

The Patient-centered Outcomes Research Institute (PCORI) has developed a network of clinical data research networks, PCORnet, whose infrastructure provides rapid, automated data extraction from the EHR and standardized data governance.^10,11^ PCORnet members extract specified data elements from their EHRs and transform them into an interoperable common data model (a way of organizing data into a standard structure), facilitating analyses across health systems.^12^ At the time of this study, PCORnet included 13 health system clinical data research networks, 20 patient-powered research networks, and 2 health plan research networks. Together, these networks included 348 health systems and more than 857,000 care providers.

To test the use of data from PCORnet members to compute benchmarks for quality of care, we focused on 3 diverse pediatric health conditions: screening children with sickle cell anemia for stroke risk, screening children taking antipsychotic medications for metabolic syndrome, and treating otitis media with appropriate antibiotics. We selected these conditions due to high prevalence, high risk of failed screening, and their use of different data types (eg, laboratories, prescriptions, etc.). These measures also rely on discrete utilization data that may be better captured in the EHR and carried forward into the PCORnet Common Data Model than criteria such as symptom severity, chronicity, functional impact, or surgical approach.^9^ National Health Lung and Blood Institute guidelines recommend using transcranial Doppler (TCD) imaging to prevent stroke in patients with sickle cell anemia. However, only 25% of eligible children in Maryland received annual screening in one study.^13,14^ Similarly, antipsychotic medication use poses long-term risks, including weight gain, diabetes, and hyperlipidemia.^15,16^ Although metabolic screening is recommended annually for patients on antipsychotics, only 27% of patients on antipsychotics insured by Medicaid in 3 states received glucose testing, and 10% received lipid testing.^17,18^ In younger children, acute otitis media (AOM) is highly prevalent and is a primary driver of antibiotic usage in pediatrics. Widely endorsed guidelines recommend using amoxicillin as a first-line antibiotic for AOM. In a study of private practices in St Louis, only 70% of eligible patients received amoxicillin (allergy rate 5%).^19,20^ For each of these conditions, there is no widely produced and validated pediatric benchmark available. Identifying top performers helps in the dissemination of best practices to lower-performing sites.^21^ NQF has developed a detailed process for specifying and testing clinical quality measures intended to address this gap.^22^ Most such measures, however, depend on manual chart abstraction. We hypothesized that the EHR-derived data standardized to the PCORnet Common Data Model provides an appropriate substrate for rapid, valid, and comparable evaluation of quality across multiple health systems to inform benchmarking and continuous improvement. To test this hypothesis, we evaluated three NQF-endorsed measures quantifying compliance with treatment guidelines in children using EHR-derived data.

## METHODS

This 2015 retrospective cohort study of children cared for in the outpatient setting included the emergency departments in 13 pediatric health systems from 2 PCORnet networks, PEDSnet and OneFlorida. We developed electronic specifications using the PCORnet Common Data Model that align with the NQF measures for (1) TCD screening among children with sickle cell anemia; (2) metabolic screening for children on antipsychotic medications; and (3) appropriate first-line antibiotics for children with AOM. We assessed the accuracy of each electronic measure compared to a manual chart review.

### Setting

PEDSnet is a network of 8 children’s hospitals, and OneFlorida is a statewide clinical research network comprising 11 academic and nonacademic health systems, including 2 Children’s hospitals.^23,24^ Together, in 2015, these networks provided care to 6 million pediatric patients. Cincinnati Children’s Hospital Medical Center was the single IRB for PEDSnet sites, and the University of Florida was the single IRB for OneFlorida sites. Both networks had existing internal data use agreements; we established a data use agreement between the lead site at OneFlorida and the data coordinating center for PEDSnet.

### Measures

We used version 3.1 of the PCORnet Common Data Model. Patient-level data drawn directly from the EHRs are mapped to a common structure (ie, same variable name, definition, etc.). Standard terminologies and coding systems used in the PCORnet Common Data Model included International Classification of Diseases 9-CM, 10-CM, and Systematized Nomenclature of Medicine, Clinical Terminology for diagnoses, Current Procedural Terminology and Healthcare Common Procedure Coding System for procedures, and Logical Observation Identifiers Names and Codes for laboratory results. The NQF algorithms for each measure were as follows: (1) “The percentage of children 2–15 years old with sickle cell anemia (Hemoglobin SS) who received at least one TCD screening within a year.”^25^ (2) “The percentage of children 1–17 years old who had 2 or more antipsychotic prescriptions and had metabolic testing (glucose and cholesterol tests).”^25^ (3) “The proportion of encounters at which antibiotics prescribed to children 2 months to 12 years old for AOM conform to the AAP/AAFP recommendation for the first-line use of amoxicillin.”^26^ We developed technical specifications based on the publicly available NQF documentation, which specifies numerators, denominators, and codes for each measure.

The data coordinating center distributed SAS queries that broadly identified potentially eligible patients and corresponding pertinent clinical data to each site. Sites securely transferred a limited dataset back for analysis. Each site maintained a crosswalk linking study codes to patient identifiers.

We used analytic code in R (R Studio, Boston, Mass.) to evaluate the measure based on the technical specifications.^27^ We found that custom laboratory orders in health systems were not associated with NQF measure specifications. Therefore, we expanded the initial code sets by searching internal EHR labels for test orders for strings matching “glucose” and “cholesterol” and reviewing the output for accuracy. We reviewed the antipsychotic medication code set with a child psychiatrist (M.S.) and updated codes to include all antipsychotic medications. At different sites, medication data contained either exclusively dispensing or prescribing events, or a combination of both. To account for a prescription followed by a dispensing event for the same drug, or multiple administrative entries for the same prescription in EHRs, we counted any prescription or dispensing event that occurred within a 15-day window as a single prescription.

### Chart Review

Seven sites from PEDSnet and 2 from OneFlorida participated in manual chart review to assess the accuracy of measures. At each site, we randomly selected an equal number of charts for patients who passed and who failed the measure; the total number of charts per site varied based on the size of their data contribution and staff availability. First, the chart reviewer assessed measure eligibility and outcome, blinded to the result of the PCORNet electronic measure evaluation. Second, we provided the electronic measure result, and the reviewer answered open-ended questions about reasons for any differences between the manual chart review and the electronic measure result. We trained chart reviewers using a didactic webinar that reviewed study background and objectives, the chart review form, and manuals, as in our prior research.^28,29^ To ensure consistency between sites, we shared answers to any questions raised by individual chart reviewers with reviewers at all sites.

### Analysis

There were 2 sites (sites L and M) that could not participate in the TCD evaluation because they could not contribute TCD screening data to PCORnet at the time of the study. Similarly, 6 sites (sites G, H, J, M, N, and O) were not able to contribute laboratory data at the time of the study, so we removed them from the metabolic screening analysis.

We calculated the rates of compliance with the NQF guideline among eligible patients for each institution. We also calculated the accuracy of each measure using a manual chart review. We did not attempt to identify whether patients were seen at more than one of the included centers because: (1) most sites are widely spaced geographically, making it unlikely that a patient would receive ongoing care in more than one location; and (2) this is not consistent with the NQF specifications.

## RESULTS

We identified 1,144 children with sickle cell anemia that qualified for the TCD measure at the 11 sites, 7,278 children on antipsychotic medications that qualified for the metabolic screening measure at 9 sites, and 49,082 children with 1 or more visits for AOM that qualified for the appropriate antibiotics measure at 11 sites (Table 1).

**Table 1.** Race and Ethnicity of the Patient Population Eligible for the 3 NQF Measures We Studied among Children Receiving Care at PEDSnet and OneFlorida Centers

Demographic	TCD Measure (N = 1,144 at 11 Sites), N (%)	Antipsychotic Measure (N = 7,278 at 9 Sites), N (%)	Otitis Media Measure (N = 49,082* at 11 Sites), N (%)
Race			
Black/African American	1,055 (92%)	1175 (16%)	10,861 (16%)
White	22 (2%)	5201 (71%)	27,116 (41%)
Asian	0	95 (1%)	1,342 (2%)
Multiple races	14 (<1%)	303 (4%)	1,741 (3%)
American Indian/Alaskan native	0	12 (<1%)	63 (<1%)
Native Hawaiian/Pacific islander	0	9 (<1%)	46 (<1%)
Other	35 (3%)	230 (3%)	3,054 (5%)
Declined to report	1 (<1%)	28 (<1%)	138 (<1%)
Unknown/no information available	17 (<1%)	213 (3%)	4,172 (10%)
Ethnicity			
Non-Hispanic	990 (87%)	6,650 (91%)	41,506 (85%)
Hispanic	40 (3%)	410 (6%)	6,508 (13%)
Other	1 (<1%)	41 (<1%)	155 (<1%)
Unknown/no information available/decline to answer	113 (10%)	177 (2%)	913 (2%)

*First encounter only.

### Site Performance on Measures Using Data Drawn from the EHR

**Fig. 1.**
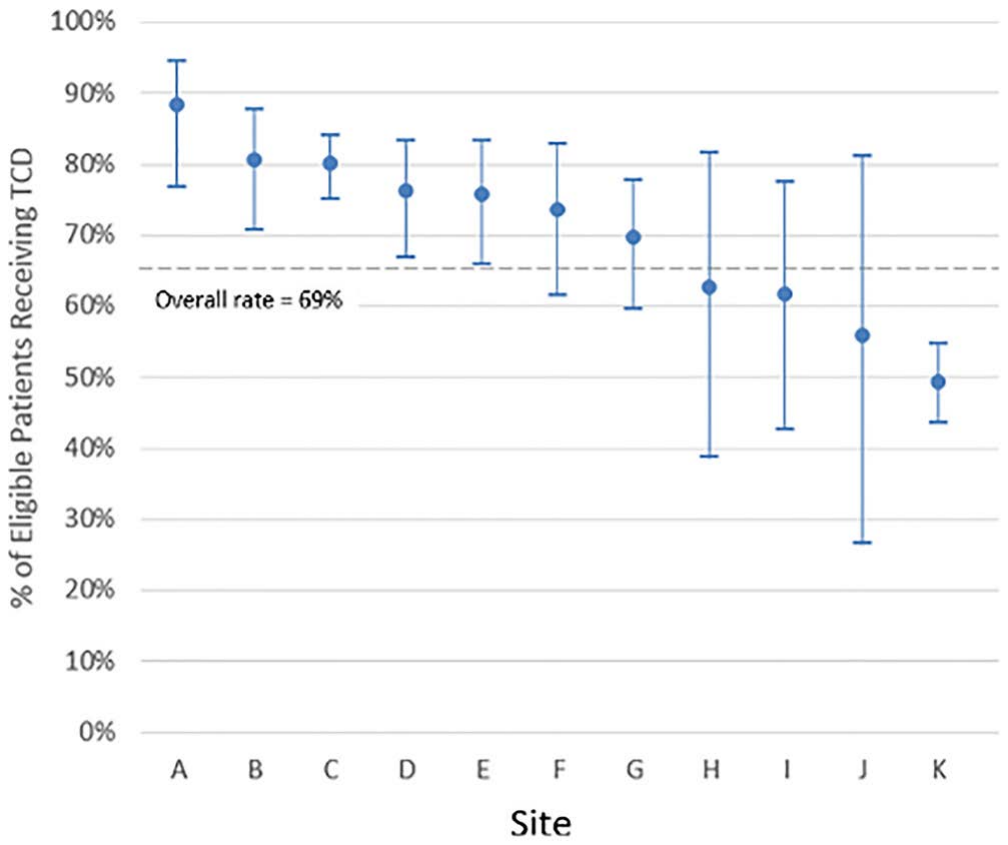
The percentage of eligible patients with sickle cell anemia receiving TCD.

Sixty-nine percent (N = 793) of eligible children received a TCD test in 2015 (range among sites: 49%–88%) (Table 2). Sites A, B, and C had screening rates of 88%, 81%, and 80%, respectively, substantially better than average (Fig. 1A). Thirty-nine percent (N = 2,923) of eligible children on antipsychotic medications received the indicated glucose and cholesterol testing (range among sites: 20%–54%). Sites E, D, and A performed substantially better than average, with screening rates of 53%, 53%, and 52%, respectively (Fig. 1B). Although we identified 67,173 eligible visits for AOM, manual chart review raised concerns about this data’s accuracy.

**Table 2.** NQF Measure Values Using PCORnet Common Data Model and Predictive Value of the Estimates of the Rates of Screening Using Manual Chart Review

Condition	Site	# Eligible Patients	Measure Value (%)	# Charts reviewed	PPV* (95% CI)	NPV† (95% CI)
TCD	A	51	88.2	18	100%	89% (52%–99%)
	B	82	80.5	41	96% (80%–99%)	88% (62%–98%)
	C	308	79.9	18	100%	89% (52%–99%)
	D	100	76.0	18	100%	89% (52%–99%)
	E	90	75.6	44	100%	95% (74%–99%)
	F	64	73.4	31	100%	69% (41%–89%)
	G	95	69.5	18	100%	100%
	H	16	62.5	16	90% (56%–99%)	83% (36%–99%)
	I	26	61.5	0	No	n/a
	J	9	55.6	0	n/a	n/a
	K	303	49.2	18	100%	55% (21%–86%)
AP	E	2127	53.7	50	100%	100%
	D	329	53.5	18	90% (56%–99%)	89% (52%–99%)
	A	1294	52.3	18	100%	78% (40%–97%)
	F	2	50	0	n/a	n/a
	I	349	33.8	0	n/a	n/a
	B	109	31.2	50	59% (33%–82%)	90% (70%–99%)
	C	1,930	27.9	9	100%	100%
	K	474	24.3	18	100%	56% (21%–86%)
	L	664	19.6	24	100%	62% (32%–86%)

*PPV indicates the predictive value of the electronic measure stating that the TCD test or laboratory result was complete compared to manual chart review results.

†NPV indicates the predictive value of the electronic measure stating that TCD test or laboratory result was not complete compared to manual chart review.

### Accuracy Compared to Chart Review

When the electronic measure identified patients as having received the TCD test in 2015, a manual review of 222 charts across eight sites agreed 98% of the time [95% confidence interval (CI): 94%–100%; range among sites 90%–100%] (Table 2). When the electronic measure identified patients as not receiving the test, chart review agreed 89% of the time (95% CI: 82%–95%; range among sites 55%–100%). The lower negative predictive validity at some sites, such as site K, occurred when TCD performed at facilities outside of the participating health system and scanned into the chart did not appear in the EHR-extracted data (Table 3).

**Table 3.** Agreement between Manual Chart Review and Data Electronically Extracted from the EHR Using the PCORnet Common Data Model

Condition	Electronic Measure Result Using PCORnet Common Data Model	% Agreement with Manual Chart Review	Range in % Agreement between Sites	Reason for Failure in Electronic Measure
TCD	Age	100%	100% at all sites	n/a
	Diagnosis of SCA	94% (95% CI: 90%–97%)	84%–100%	Other hemoglobinopathies (Hb SC, Hb S-Beta^+^ Thalassemia)
	TCD was performed	98% (94%–100%)	90%–100%	n/a
	TCD was NOT performed	89% (82%–95%)	56%–100%	TCD test was performed at an outside facility and scanned into chart. This did not show in the PCORnet Common Data Model
AP	>2 antipsychotic dispensing events	92% (86%–95%)	90%–100%	Dispensing more accurate
	Glucose or HbA1C test performed	100%	n/a	
	Glucose or HbA1C test NOT performed	81% (70%–89%)	62%–100%	Outside laboratories
	Cholesterol test performed	100%	n/a	
	Cholesterol test NOT performed	88% (79%–94%)	61%–100%	Outside laboratories
AOM	Age	99%	97%–100%	Age was incorrect
	Diagnosis of ear infection	100% (94%–100%)	100% at all sites	
	No antibiotics prescribed in the last 30 d	98% (95%–99%)	95-100%	Electronic measure missed antibiotic prescription
	Appropriate prescribed in the last 30 d	100% (94%–100%)	100% at all sites	
	12 visits total (5 sites) from the electronic measure were *not in the chart*	n/a	n/a	EHRs may filter visits typically considered uninteresting from display for manual chart review

SCA, sickle cell anemia.

There were 18 patients (18%) in our review of 102 charts, where the NQF measure found that TCD screening was missed for whom TCD was not clinically indicated. This finding included 5 patients across three sites with a stem cell transplant and 13 patients across 4 chronic transfusion therapy sites. Also, 6 patients across 3 sites did not have a TCD performed in 2015 but did have one in the last 2 months of 2014 or the first 2 months of 2016.

In our manual review of 155 charts at 8 sites for children on antipsychotic medications, when the electronic measure identified patients as having received the indicated metabolic screening tests in 2015, the manual review agreed 88% (95% CI: 81%–94%; range across sites 59%–100%) of the time at all sites. When the electronic measure identified patients as not receiving the test, the manual review agreed 86% (95% CI: 78%–93%; range across sites 56%–100%) of the time. At some sites, laboratory tests performed at outside facilities and scanned into the EHR did not appear in the electronic EHR-derived data, causing lower accuracy (Table 3). There were 13 instances across 3 sites where the chart did not indicate multiple prescriptions of antipsychotic medications but dispensing data in the PCORNet data indicated multiple dispensings. In this situation, we considered the PCORNet data to be more accurate than the chart and included these patients in the measure.

In our manual review of 256 visits at 8 sites, electronic measure AOM diagnoses agreed with the chart 100% of the time at all sites (Table 3). However, the electronic measure did not identify 35 AOM visits. This result was because the NQF measure definition did not include frequently use AOM visit codes. There were also 12 visits noted by the electronic measure not identified in chart review, potentially because EHRs may filter visits typically considered uninteresting from the display for manual review.

## DISCUSSION

We found that using data drawn directly from the EHR was both accurate and efficient for benchmarking healthcare quality. In implementing 3 pediatric NQF measures, we found several opportunities for improvement to the measures. Improvement in the quality of care is needed in all three measures assessed among the six million children cared for at these 13 sites.

This study identified some opportunities for improvement in each of the 3 pediatric NQF measures of the study. The TCD measure among children with sickle cell anemia was easy to implement, and the electronic measure using EHR-derived data had good accuracy compared to chart review. However, the measure identified some patients, such as those with bone marrow transplants, as not receiving a needed TCD when, in fact, the TCD was not indicated. The NQF code set excluded some laboratory orders for metabolic screening at each site. This measure was also developed using Medicaid analytic files; to assess care quality by a particular health system, we assumed that both antipsychotic prescribing and laboratory testing occurred at the same health system. This assumption excluded patients receiving prescriptions from a psychiatrist in the community but medical care at the health system. Finally, a commonly used diagnostic code, “otitis media not otherwise specified,” was not included in the NQF measure specifications, leading to several missed AOM visits.

We found using data drawn from the EHR through the PCORnet Common Data Model to be highly efficient and accurate. Standardization of data definitions and use across all sites allowed for interoperability. The incremental cost of refining measures was low, allowing iterative improvements and tailoring of measures. The PCORnet study governance structure is highly efficient; it took under 2 months from IRB approval at the lead site to approval at all sites in both networks. Compared to publications using NQF measures based on billing data, we detected more qualifying patients. We selected these measures specifically because they relied on discrete utilization data that we expected to be captured better in the EHR and carried forward into the PCORnet CDM than criteria such as symptom severity, chronicity, functional impact, or surgical approach.^9^ This has been a motivating principle of the Meaningful Use initiative standards and Common Data Models such as the PCORnet, OMOP,^30^ and Sentinel^31^ models. These models are designed to capture utilization events accurately. Within this ecosystem, EHR implementers can take steps to ensure utilization events are available for capture, such as: to ensure that critical events are captured within the EHR, rather than being shunted to other business processes; maintain accurate mappings from local codes and terms used to manage workflow and the standardized terminologies that enable multisite measurement; and to develop accurate and automated processes for transformation of local data into Common Data Models, to facilitate development and testing of measures against them, to establish a reliable base of information.

However, there was variation in the speed that sites could adjust to upgrades in the data reported to the Common Data Model, causing some sites to not report some data for this study. Also, the PCORnet Common Data Model could not detect scanned documents, including test results, in the EHR. To address problem with access to textual records, including scanned documents, there are initiatives in the clinical informatics community to approach feature extraction methods such as natural language processing systematically. Alternative approaches integrate discrete data capture better into clinical workflows or adopt registry strategies for population management that better account for primary data (eg, querying for HbA1c levels directly). The NQF e-Measure standards represent a deliberate effort to encourage and standardize such practices.

For each of the measures we studied, improvements are needed in most sites’ quality of care. To date, there are very few studies comparing rates of screening in each of these areas nationally. For example, for TCD screening, a *manual review* of 3,539 charts at 9 sites, in follow-up to the STOP trial, found TCD screening rates of 18%–91%.^32^ In Tennessee Medicaid Claims, the TCD screening rate was 68%.^33^ Older and single-site studies estimate rates of annual TCD screening from 25% to 45%.^13,34^ Although the overall screening rate in our study was only 69%, the best site achieved a screening rate of almost 90%. Multiple sites performed substantially better than average. The ease of using electronic data collection methods, drawn directly from the EHR, can facilitate the regular weekly or monthly data collection needed to test quality improvement interventions to improve screening. Using quality improvement methods for screening among 2 year olds, one sickle cell center increased the first-screening rates to 100% using quality improvement methods.^35^

The number of children taking antipsychotic medications has skyrocketed in the past decade, to 629, 914 children in 2010.^36,37^ These children have 4 times the risk of diabetes and increased cholesterol.^16^ Previously published annual glucose screenings ranged from 13% to 30%, and annual cholesterol screening ranged from 17% to 30%.^18,38–40^ In our study, only 38.5% of children received indicated metabolic screening tests annually.

For both of these measures, there were hospital sites that performed substantially better than average and sites that performed substantially worse than average. Informed by our data, in collaboration, sites could identify best practices to improve the quality of care nationally. This model has been effective among pediatric quality improvement collaboratives, such as the Solutions for Patient Safety Network, a network of over 135 hospitals working together to eliminate harm to children caused by healthcare.

This study suggests that using EHR-derived data, as in the PCORnet Common Data Model, is a useful tool for benchmarking healthcare quality. The accuracy of the electronically extracted data was high, with substantially less labor required to produce than chart abstraction. With the gap between desired performance and observed metrics and wide variations between sites observed, the measures are sufficiently precise to inform quality improvement activities and identify best practices informed by benchmarking. Recent work also suggests that parents and health system leaders support using such measures to support better awareness and decision-making surrounding appropriate care.^41^ The NQF has an active program for measure development, including electronic measures. Our work demonstrates few barriers in a large-scale network like PCORnet to implementing such a scheme to improve healthcare quality assessment.

## DISCLOSURE

The authors have no financial interest to declare in relation to the content of this article.

## ACKNOWLEDGMENTS

We acknowledge Carla McGraw for her thorough and thoughtful feedback on the chart review forms and methods. We also thank Rita Mangione-Smith, Kelly Kelleher, Amanda Dempsey, Peter Margolis, Tim Wysocki, and Karen Leonhart for their support of the study.
